# *Starship* giant transposable elements cluster by host taxonomy using *k*-mer-based phylogenetics

**DOI:** 10.1093/g3journal/jkaf082

**Published:** 2025-04-11

**Authors:** Rowena Hill, Daniel Smith, Gail Canning, Michelle Grey, Kim E Hammond-Kosack, Mark McMullan

**Affiliations:** Earlham Institute, Norwich, Norfolk NR4 7UZ, UK; Intelligent Data Ecosystems, Rothamsted Research, Harpenden, Hertfordshire AL5 2JQ, UK; Protecting Crops and the Environment, Rothamsted Research, Harpenden, Hertfordshire AL5 2JQ, UK; Earlham Institute, Norwich, Norfolk NR4 7UZ, UK; Protecting Crops and the Environment, Rothamsted Research, Harpenden, Hertfordshire AL5 2JQ, UK; Earlham Institute, Norwich, Norfolk NR4 7UZ, UK

**Keywords:** cargo-mobilizing elements, Gaeumannomyces, Ascomycota, Pezizomycotina, Fungi

## Abstract

*Starships* are a recently established superfamily of giant cargo-mobilizing transposable elements in the fungal subphylum *Pezizomyotina* (phylum *Ascomycota*). To date, *Starship* elements have been identified up to ∼700 kbp in length and carry hundreds of accessory genes, which can confer both beneficial and deleterious traits to the host genome. Classification of *Starship* elements is centered on the tyrosine recombinase gene that mobilizes the element, termed the captain. We contribute a new perspective to *Starship* relatedness by using an alignment-free *k*-mer-based phylogenetic tree-building method, which can infer relationships between elements in their entirety, including both active and degraded elements and irrespective of high variability in element length and cargo content. In doing so we found that relationships between entire *Starships* differed from those inferred from captain genes and revealed patterns of element relatedness corresponding to host taxonomy. Using *Starships* from root/soil-dwelling *Gaeumannomyces* species as a case study, we found that *k*-mer -based relationships correspond with the similarity of cargo gene content. Our results provide insights into the prevalence of *Starship*-mediated horizontal transfer events. This novel application of a *k*-mer -based phylogenetics approach overcomes the issue of how to represent and compare highly variable *Starship* elements as a whole, and in effect shifts the perspective from a captain to a cargo-centered concept of *Starship* identity.

## Graphical Abstract

**Graphical Abstract:**
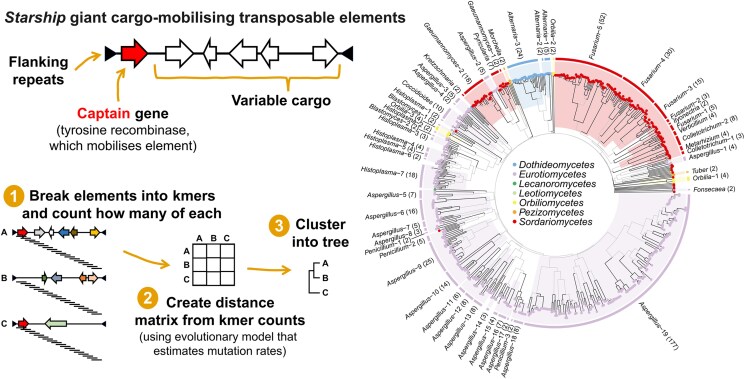

## Introduction

Transposable elements (TEs), or transposons, are stretches of DNA, typically between 100 and 10,000 bp in length, which can independently move and replicate within the genome (Biémont 2010; Wells and Feschotte 2020). Thanks to advances in long-read sequencing, highly contiguous genome assemblies have revealed the existence of TEs hundreds of kilobases in length (Arkhipova and Yushenova 2019). Some of these large TEs have been shown to harbor both genes necessary for their mobilization as well as miscellaneous accessory genes, and are accordingly referred to as cargo-mobilizing elements (CMEs; Gluck-Thaler and Vogan 2024). Recently, giant CMEs have been found in various species in the fungal subphylum *Pezizomycotina* (phylum *Ascomycota*; McDonald *et al*. 2019; Vogan *et al*. 2021; Urquhart *et al*. 2022), and have since been determined to belong to a newly established TE “superfamily” (sensu Wicker *et al.* (2007)) or “subclass” (sensu Wells and Feschotte (2020)) known as “*Starships”* (Gluck-Thaler *et al.* 2022). To date, *Starship* CMEs have been found to range in length from 15 Kbp (Gluck-Thaler *et al.* 2024) to ∼700 kbp (Urquhart *et al.* 2024).

*Starship* mobilization is mediated by a leading 5′ located gene containing the DUF3435 domain (protein family accession PF11917), termed the “captain,” which encodes a tyrosine recombinase that initiates movement of the TE into a new genomic location via a “cut-and-paste” mechanism (Urquhart *et al.* 2023). This is similar to the hypothesized mobilization process of the “*Crypton”* class II DNA transposon superfamily (Wells and Feschotte 2020), which was incidentally also first discovered in fungi (Goodwin *et al.* 2003), although this TE superfamily has since been found in other eukaryotes (Kojima and Jurka 2011). Tyrosine recombinase domains in *Starship* captain genes and *Cryptons* are very distantly related (Gluck-Thaler *et al.* 2022) and, unlike *Cryptons*, *Starship* elements are sometimes flanked by tandem inverted repeats (TIRs) in addition to direct repeats (DRs), and can contain a highly variable and often sizeable cargo of accessory genes (Gluck-Thaler and Vogan 2024). *Starship* cargos can harbor genes that are beneficial to the fungus, for example those associated with plant virulence (McDonald *et al.* 2019), metal tolerance (Urquhart *et al.* 2022), and climate adaptation (Tralamazza *et al.* 2024). However, as selfish genetic elements, *Starships* may also mobilize cargo that is neutral or even detrimental to the overall fitness of the host genome (Vogan *et al.* 2021).

Classification within the *Starship* CME superfamily is focused on the captain gene, using both phylogenetic relationships between captain genes to define “family” and ortholog clustering of captain genes to define “navis” (i.e. a ship) (Gluck-Thaler and Vogan 2024). Both the captain family and the flanking DRs are thought to influence the genomic site that an element is inserted into, with *Starships* of certain captain families preferentially inserting into, for instance, other TEs or 5S rDNA (Urquhart *et al.* 2023; Gluck-Thaler and Vogan 2024). DUF3435-containing tyrosine recombinase genes are more usually found “solo,” rather than within a cargo-carrying element, i.e., as a captain; however, it is not clear to what extent this is due to the failure to detect the boundaries of an element or because pseudogenization of the tyrosine recombinase gene has occurred (Gluck-Thaler and Vogan 2024). *Starship* captain genes do not form a single monophyletic cluster in the DUF3435 tyrosine recombinase gene tree and are instead scattered across the phylogeny amongst other apparently “solo” DUF3435-containing tyrosine recombinase genes (Gluck-Thaler *et al.* 2022; Hill *et al.* 2025). Due to their highly divergent nature, tyrosine recombinase gene sequences are also difficult to align, introducing uncertainty into conventional alignment-based phylogenetic analyses. It is not currently possible to determine whether these relationships described by captains are preserved or representative of the *Starships* as a whole, considering that elements are highly variable in terms of cargo and overall length. This also limits phylogenetic assessment of the prevalence of (or boundaries to) horizontal exchange across the *Pezizomycotina*. In an effort to represent distinction in cargo content, Gluck-Thaler and Vogan (2024) introduced the additional definition of “haplotype,” based on clustering of *k*-mer similarity scores. Here, we have taken this approach 1 step further and used a *k*-mer -based phylogenetic tree-building method to contribute a new perspective to *Starship* relatedness. In doing so, we have revealed previously obscured patterns of *Starship* relatedness corresponding to host taxonomy.

To determine whether the relatedness revealed by the *k*-mer trees conformed with similarity in cargo gene content, we explored the cargos of *Starships* previously identified from genomes within the genus *Gaeumannomyces* (Hill *et al.* 2025). This genus comprises soil-dwelling fungi that are also both pathogenic and nonpathogenic wheat and wild grass root associates (Palma-Guerrero *et al.* 2021; Chancellor *et al.* 2024). These elements provided an ideal case study as they vary greatly in overall size and number of cargo genes within their host taxonomy clusters. The genomes were also all generated in parallel using the same long-read sequencing technology and a cross-referent annotation pipeline (Hill *et al.* 2025). Given the impact of assembly and annotation quality on *Starship* recovery (Gluck-Thaler and Vogan 2024), these *Gaeumannomyces* elements therefore represent a consistent dataset that is impacted to a lesser extent by the technology used to produce them.

## Materials and methods

### *K*-mer -based phylogenetic analysis

To compare phylogenetic reconstruction of whole elements vs captain genes, we used a curated set of 39 *Starships* from Gluck-Thaler *et al.* (2022) and Gluck-Thaler and Vogan (2024) alongside 14 *Gaeumannomyces Starships* predicted using the tool starfish v1.0.0 (Gluck-Thaler and Vogan 2024) in our previous study (Hill *et al.* 2025). Only *Gaeumannomyces Starships* with predicted flanking repeats were used. We used entire element sequences as input for the *k*-mer -based method Mashtree v1.4.6 (Katz *et al.* 2019) with 1,000 bootstrap replicates and the –min-depth 0 parameter to discard very unique *k*-mers, recommended to improve accuracy. We used the corresponding captain genes as input for a maximum likelihood (ML) tree, first aligning gene sequences using MAFFT v7.271 (Katoh and Standley 2013), trimming using trimAl v1.4.rev15 (Capella-Gutiérrez *et al.* 2009), and finally building the ML tree using RAxML-NG v1.1.0 (Kozlov *et al.* 2019) with bootstrapping until convergence, which occurred after 150 bootstrap replicates. We visualized concordance between the 2 phylogenies via a tanglegram, produced in R v4.3.1 (R Core Team 2023) using the packages ape v5.7-1 (Paradis and Schliep 2019), phytools v2.1-1 (Revell 2024) and ggtree v3.9.1 (Yu *et al.* 2017). We calculated the normalized Robinson–Foulds (RF) distance between the element and captain phylogenies using the RF.dist function from the phangorn v2.7.0 package (Schliep *et al.* 2017).

We then used a larger dataset of *Starships* predicted using the tool starfish v1.0.0 by Gluck-Thaler and Vogan (2024) to assess whether patterns in the curated *k*-mer tree would persist with broader sampling. Comparisons were made using the entire dataset including elements without predicted flanking repeats (597 elements + 20 *Gaeumannomyces* elements = 617 total) against a filtered dataset of only elements with predicted flanking repeats (343 elements + 14 *Gaeumannomyces* elements = 357 total) to explore the impact of uncertain element boundaries on the topology. For both cases, entire element sequences were again run with Mashtree, but with 100 bootstrap replicates and the default –min-depth parameter to accommodate for the much larger dataset. Previously determined *Starship* family classifications, based on captain phylogenetic relationships (Gluck-Thaler and Vogan 2024), were mapped to element *k*-mer tree tips to visualize the distribution of families across clades using the additional R packages ggtreeExtra v1.10.0 (Xu *et al.* 2021) and glottoTrees v0.1.10 (Round 2021).

Mashtree estimates similarity between *k*-mer sketches using the Mash distance, which models mutation rates under a simple Poisson process of random site mutation (Ondov *et al.* 2016). To compare this with an alternative evolutionary model we used sourmash v4.8.14 (Irber *et al.* 2024) to calculate a distance matrix with the --estimate-ani parameter. Like the Mash distance, average nucleotide identity (ANI) as implemented in sourmash is computed from the Jaccard index, but unlike Mash it does not make the assumption that all *k*-mers are mutated independently, which can result in Mash overestimating mutation rates (Rahman Hera *et al.* 2023). The *k*-mer sketching algorithm within sourmash, FracMinHash, may also outperform Mash's MinHash algorithm when used on very different set sizes (Rahman Hera *et al.* 2023). We should caveat that ANI was developed for use with prokaryote data and has not, to our knowledge, been validated with eukaryote data, although this may predominantly be due to scalability issues when working with larger eukaryote genomes. We used the ape nj command in R to generate a neighbor-joining tree from the sourmash ANI distance matrix, which is conceptually the same tree-building approach that is integrated into Mashtree.

### Exploration of cargo gene content in *Gaeumannomyces* elements

We used the aforementioned larger dataset of 20 *Starships* predicted from 7 *Gaeumannomyces* genomes to assess whether similarities in cargo gene content corresponded with the patterns of relatedness described by the *k*-mer trees. We characterized orthologous genes predicted in our previous study (Hill *et al.* 2025) as being core, accessory, or specific within the set of 20 elements, and their sharedness was visualized using the R package ComplexUpset v1.3.3 (Krassowski 2022). After normalizing cargo orthogroup presence-absence values with the base R scale function, we produced a Euclidean distance matrix using the R dist function and performed hierarchical clustering with the hclust function using the “complete” agglomeration method. We then compared the topology produced by hierarchical clustering with phylogenetic relationships from the larger *k*-mer -based tree using a tanglegram and calculated the normalized RF distance, as described above. We also determined the location of cargo orthogroups—i.e. whether orthologous genes were only found inside elements or also found in the wider genome.

We searched for specific genes or domains previously reported to be prevalent in *Starships* or with assigned functional roles of particular note (Gluck-Thaler *et al.* 2022) using BLAST v2.10 (Camacho *et al.* 2009) and also PFAM domain assignment from the functional annotation (Hill *et al.* 2025). Namely: DUF3723, ferric reductase (FRE), patatin-like phosphatase (PLP), ToxA effector, spore killing (Spok) genes, and associated domains. We additionally made BLAST searches against the Pathogen–Host Interactions Database v4.17 (PHI-base; (Urban *et al.* 2025) downloaded on August 1, 2024, and considered a positive match when at least 50% of genes in an orthogroup had the same hit. We assessed whether Gene Ontology (GO) terms were enriched amongst cargo genes using the R package topGO v2.52.0 (Alexa and Rahnenfuhrer 2022) with Fisher's exact test and the weight01 algorithm.

In addition to previously mentioned packages, data analysis and visualization were performed using the following R packages: cowplot v1.1.3 (Wilke 2024), ggforce v0.4.2 (Pedersen 2024), gggenomes v1.0.0 (Hackl *et al.* 2024), ggnewscale v0.4.10 (Campitelli 2024), ggpubr v0.6.0 (Kassambara 2023), ggrepel v0.9.5 (Slowikowski 2024), matrixStats v1.3.0 (Bengtsson 2024), patchwork v1.2.0 (Pederson 2024), scales v1.3.0 (Wickham and Seidel 2023), tgutil v0.1.15 (Chomsky and Lifshitz 2023), and tidyverse v2.0.0 (Wickham *et al.* 2019).

## Results and discussion

### A *k*-mer -based approach for *Starship* phylogenetics recovers signal corresponding to host taxonomy

**Fig. 1.**
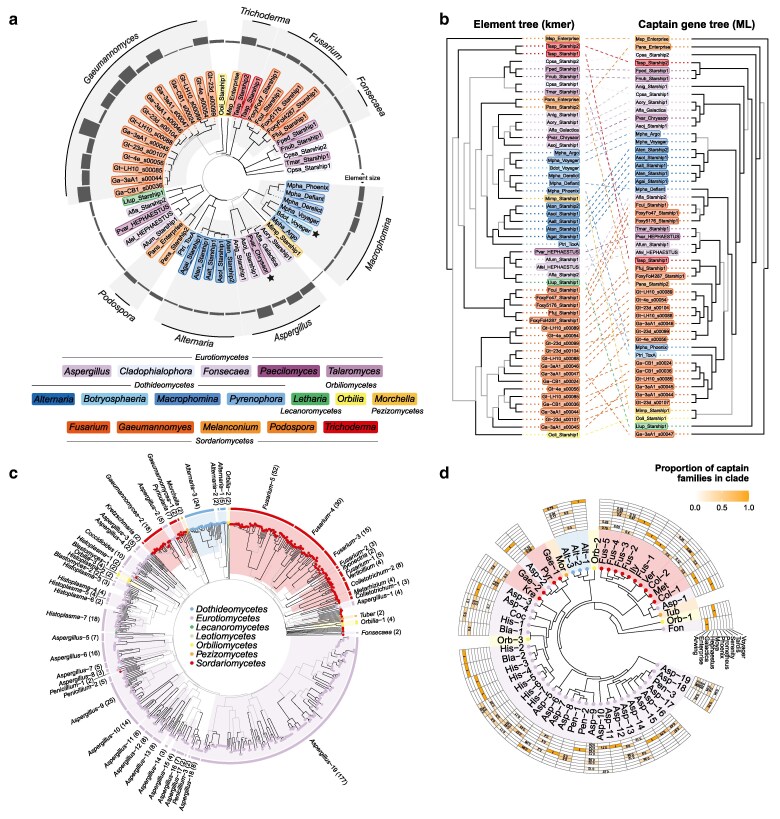
*K*-mer-based phylogenetic analyses of *Starship* elements. a) An unrooted *k*-mer-based phylogenetic tree of 53 *Starships*—39 curated elements from 33 *Pezizomycotina* species (Gluck-Thaler *et al.* 2022; Gluck-Thaler and Vogan 2024) and 14 predicted by starfish from *Gaeumannomyces* species (Hill *et al.* 2025). Gray branches indicate bootstrap support < 70. Tip points are colored by genus and the outer ring indicates total element length. Black stars beside tips highlight elements from another genus in an otherwise monophyletic clade. b) A tanglegram comparing the topology of the *k*-mer-based element tree in a) and a maximum likelihood gene tree of the corresponding captain genes (see Supplementary Fig. 1 for the unrooted captain tree). Both trees are arbitrarily rooted with the Msp_Enterprise element. Gray branches indicate bootstrap support < 70. c) An unrooted *k*-mer-based phylogenetic tree of 617 *Starships* predicted with starfish (Gluck-Thaler and Vogan 2024; Hill *et al.* 2025), with gray branches indicating bootstrap support < 70. Genus-level monophyletic clades are highlighted and labeled, with the number of elements in each clade shown in brackets. Clades and tips are colored by host taxonomic class. See Supplementary Fig. 2 for element tip labels and captain-based family classifications. d) A summary of the *k*-mer -based tree in c) with genus-level monophyletic clades collapsed. The outer grid summarises *Starship* family classifications based on captain genes for the elements in each clade, with a darker grid cell colour indicating a higher proportion of the elements within the clade belonging to that family. Clades with no grid cells did not have any classified captain data.

We used a *k*-mer -based approach for phylogenetic analysis of *Starships* to produce a phylogenetic tree of 53 entire *Starship* element sequences from Gluck-Thaler *et al*. (2022) and Hill *et al*. (2025), encompassing 17 host genera across 6 classes in the *Pezizomycotina*. We found elements to broadly cluster by genus, even when differing greatly in length (Fig. 1a). This contrasted with the captain gene tree (Supplementary Fig. 1) and element and captain trees were frequently discordant (RF distance 0.73 = 73% differing bipartitions; Fig. 1b), i.e. *Starships* that were more closely related according to their *k*-mer profiles could have very divergent captain genes. There were some exceptions to element/captain discordance; for instance, similar relationships in both captain and element trees were observed for the *Alternaria* clade (Fig. 1b). *Alternaria* captains were also closely related to some *Macrophomina* captains, in reflection of expected host species relationships in the *Dothideomycetes*; however, dothideomycete captains were not monophyletic as *Macrophomina* captains were also dispersed across other clades in the captain tree (Supplementary Fig. 1). Overall, 6/10 host genera with more than 1 genome represented were monophyletic in the element tree vs 2/10 in the captain tree. Also note the placement of Mpha_Derelict—a previously “unclassifiable” deactivated *Starship* missing the captain gene—alongside other elements from *Macrophomina* species (Fig. 1a). Two striking disruptions of this host clustering were caused by the elements Bdot_Voyager and Pvar_Chrysaor, the latter of which has been recently asserted to be horizontally transferred between various eurotiomycete species (Urquhart *et al.* 2024).

To determine if these observations of clustering by host taxonomy extended more broadly across the *Pezizomycotina*, we used the same *k*-mer -based phylogenetics method on a larger dataset of 597 elements systematically predicted using the tool starfish by Gluck-Thaler *et al*. (2024) alongside 20 *Gaeumannomyces* elements (Hill *et al.* 2025). This again recovered widescale clustering by host taxonomy, with the additional clear formation of clades broadly corresponding to host class level (Fig. 1c; Supplementary Fig. 2). We also performed a more conservative analysis to minimize the risk of including *k*-mers from the background genome, where we filtered the larger dataset to include only elements with predicted flanking DRs (343 elements + 14 *Gaeumannomyces* elements), which broadly reflected the results from the unfiltered dataset (Supplementary Fig. 3). As may be expected from the observed element/captain tree discordance in Fig. 1b, family classifications based on captains were scattered across the larger starfish-predicted element *k*-mer trees (Fig. 1d, Supplementary Figs. 2 and 3). The degree of element/captain phylogenetic discordance is important because phylogenetic relationships of captains have been the predominant factor in element classification (Gluck-Thaler and Vogan 2024).

**Fig. 2.**
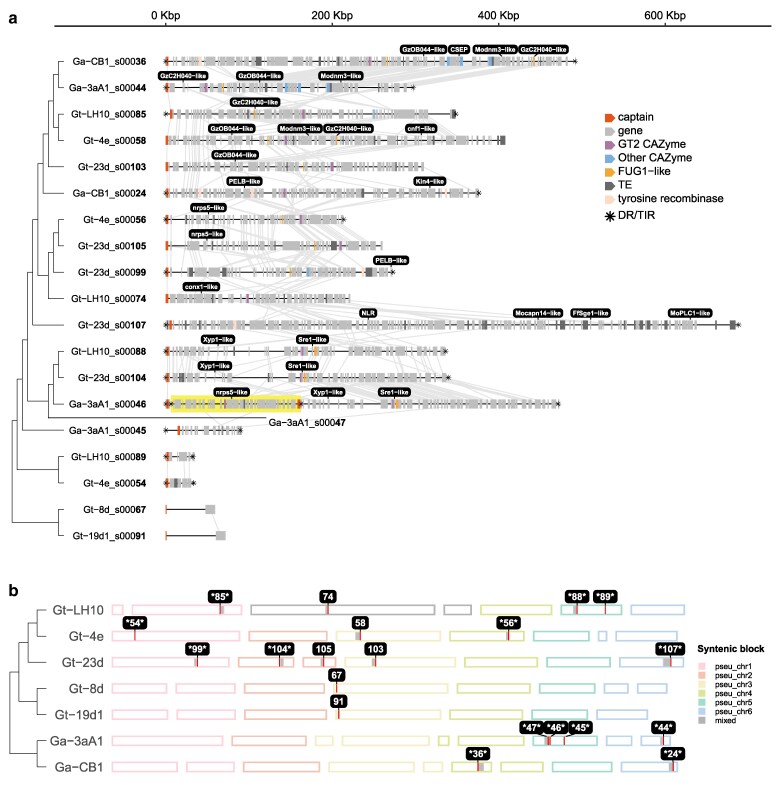
Summary of the *Gaeumannomyces Starships* predicted using starfish by Hill *et al*. (2025). a) A schematic of the 20 *Starships* ordered by phylogenetic relationships taken from Supplementary Fig. 2. Synteny between orthologous genes in neighboring elements is indicated with gray lines. A nested element (Ga-3aA1_s00047) is highlighted. Common genes are colored with known functions and the presence of flanking DRs or TIRs are indicated with an asterisk. Genes of note are labeled in black boxes. b) Ideograms showing the position of the 20 *Starships* across pseudochromosomes, adapted from Hill *et al*. (2025). ID numbers correspond to the bolded numbers for each element in a). Elements with flanking DRs are indicated with asterisks either side of the element ID number.

Phylogenetic discordance in comparison to species relationships is frequently used as evidence for horizontal gene transfer (HGT) (Ravenhall *et al.* 2015); however, there are a number of alternative biological and/or analytical factors that can also result in a similar pattern (Steenwyk *et al.* 2023). Trans-species polymorphisms, where polymorphism originates before speciation and is preserved, potentially by balancing selection, can result in genes being more similar between species than within. Trans-species polymorphisms have been reported in fungal genes associated with vegetative incompatibility (Milgroom *et al.* 2018; Auxier *et al.* 2024), and such genes have been found multiple times in *Starships* (Fig. 2a; Gluck-Thaler *et al.* 2022, 2024; Urquhart *et al.* 2024). Even without natural selection, neutral processes such as incomplete lineage sorting, recombination and gene conversion, and gene duplication and loss can elevate levels of discordance (Bjornson *et al.* 2024). The latter is a particularly aggravating factor for misidentifying HGT as it can result in paralogues being mistaken as orthologues (Smith and Hahn 2021).

Another suite of commonly used methods to detect HGT are “surrogate” phylogenetics methods, which do not build a tree but still assess evolutionary distances, e.g. using sequence similarity (Ravenhall *et al.* 2015); however, the results of surrogate methods can still be confounded by the phenomena described above. A sequence similarity approach also comes with the caveat that the best BLAST hit is not necessarily the closest related gene (Koski and Golding 2001) and requires subjective decisions about acceptable similarity thresholds. Distinguishing the cause(s) of phylogenetic discordance can be especially difficult for closely related taxa (Steenwyk *et al.* 2023), which is relevant here as elements from different host species were scattered amongst each other within genus-level clades in all *k*-mer -based tree analyses (Fig. 1a, Supplementary Fig. 2). Due to semipermeable species boundaries in fungi, interspecific hybridization within the genus level has been detected multiple times (Steenkamp *et al.* 2018; Steensels *et al.* 2021). In such cases, *Starships* could be inherited during sexual reproduction between 2 different species and subsequent backcrossing could leave the element as an introgression, which may be mistaken as having been horizontally transferred. For all the reasons outlined above, general frequency of HGT events may have been overestimated in fungi (Kurland *et al.* 2003; Dupont and Cox 2017). The *k*-mer -based phylogenetics approach described here may be useful in certain contexts as 1 piece of evidence toward identifying (or dismissing) HGT, but the confounding factors described above would need to be assessed to have confidence that HGT has occurred (e.g. Fijarczyk and Babik 2015; Knowles *et al.* 2018). A number of the above factors contributing to discordant relationships are likely to have a greater impact for more closely related species, and it may be important to focus attention on apparent HGT events across greater evolutionary distances, which are presumed to be rarer, at least in prokaryotes (Popa *et al.* 2017; Burch *et al.* 2023; Dmitrijeva *et al.* 2024).

In the larger *k*-mer -based tree there were many within genus subclades of elements with captains of the same family, but also cases where minimally diverged sister elements had different captains. For example: aspcri2_s00912 and aspcri1_s00891 from different host genomes within the *Aspergillus*-9 clade had Phoenix and Prometheus captains, respectively; and aspnig6_s01954 and aspnig6_s01955 from the same host genome within the clade *Aspergillus*-19 had Hephaestus and Phoenix captains, respectively (Supplementary Fig. 2). It should be noted that there is some uncertainty as to the boundaries of these elements, as in these cases elements did not have predicted flanking repeats. A similar observation was made by Gluck-Thaler and Vogan (2024) for *Starship* pairs with near-identical cargo “haplotypes” but different captain-derived families. Together with the fact that captain genes are phylogenetically indistinguishable from “lone” tyrosine recombinase genes harboring the DUF3435 domain (Gluck-Thaler *et al.* 2022; Hill *et al.* 2025), this prompts the question as to whether *Starships* can swap the captain for a different tyrosine recombinase gene, which would render the “captain” status as somewhat transient. A previous study has already reported that *Starship* elements can lose their captain gene to become “degraded” or “derelict” (Gluck-Thaler *et al.* 2022), and in another study a mechanism has been suggested wherein different elements partake in cargo swapping (Urquhart *et al.* 2024). A similar mechanism where the captain, as opposed to the cargo, is swapped to acquire a captain gene from a different family could be a strategy to diversify insertions of virtually identical elements into different target sites. Comparing the *k*-mer profiles of regions surrounding CMEs could incidentally be another fruitful avenue for understanding target site preference, as many *Starships* have been found to insert into other TEs and AT-rich regions but without clear patterns in, for instance, TE superfamily or domain (Gluck-Thaler and Vogan 2024).

Aside from the major clade in the larger starfish *k*-mer tree overrepresented with elements from eurotiomycete hosts, other eurotiomycete elements appeared scattered amongst other clades, although there were lower support values for deeper tree nodes (Fig. 1c). It is notable that eurotiomycete elements dominate the starfish dataset—of all the genomes explored by Gluck-Thaler and Vogan (2024), *Eurotiomycetes* was the class with the highest proportion of genomes returning a *Starship* (36%; Supplementary Fig. 4). This was closely followed by the *Orbiliomycetes* (28%), despite 16 times fewer orbiliomycete genomes having been surveyed compared with the *Eurotiomycetes*, and orbiliomycete element clades were similarly widespread across the *k*-mer tree (Fig. 1c). As one of the earliest diverging classes within the *Pezizomycotina* subphylum, the *Orbiliomycetes* are distantly related to *Eurotiomycetes* (Li *et al.* 2021), and they do not share ecological distributions more so than other taxonomic classes, so the underlying biological explanation is unclear. The far larger *Eurotiomycetes* class comprises diverse lifestyles including: rock-inhabiting fungi and other extremophiles; plant and animal pathogens; lichenized and lichen-associated fungi; ectomycorrhizal fungi; ant mutualists; and saprotrophs (Geiser *et al.* 2015). The *Orbiliomycetes* are primarily thought to be saprotrophs but include some soil-dwelling carnivorous fungi that trap invertebrates (Pfister 2015). Variation in the rate of *Starship* recovery in the genomes of different taxonomic classes could be a result of inconsistencies in assembly quality or bias within the starfish tool to recover certain elements from certain classes. However, these results do suggest that there may be a relationship between the tendency for a taxonomic class to have *Starship* elements and greater diversity of element clades.

While we consider this to be a promising application for *k*-mer -based phylogenetics, we must note that such methods were typically developed for whole-genome data. We are not aware of *k*-mer -based phylogenetic methods having been tested on sequences such as fungal CMEs. However, given that such methods are considered well-suited to viral genomes due to their high levels of mutation, gene duplication, and rearrangement (Zielezinski *et al.* 2017), CMEs would appear to be a similarly appropriate use case. Other than circumventing issues with alignment, *k*-mer -based methods also have the advantage of being more computationally efficient than alignment-based phylogenetic methods, which could reduce the carbon footprint of analyses (Grealey *et al.* 2022). There are many different approaches and tools for alignment-free sequence comparison which would warrant further testing in the context of CME phylogenetics (Luczak *et al.* 2019; Zielezinski *et al.* 2019). For instance, ANI is frequently used as a distance metric for prokaryote genomes and, as implemented in sourmash, has the benefit of a more realistic evolutionary model of mutation than that used by Mash (Rahman Hera *et al.* 2023), but whether it is appropriate for eukaryote data has yet to be validated. Nonetheless, we found that trees generated from ANI distance matrices produced using sourmash were broadly consistent with our Mashtree results (Supplementary Figs. 5 and 6) and supported our conclusion that *Starships* predominantly cluster according to host taxonomy. We were unable to produce a *k*-mer tree for captain genes using Mashtree, presumably due to the much smaller sequence length of a single gene. This meant we were not able to directly compare whole element and captain trees using the same *k*-mer -based method. However, at the genome-scale, previous comparisons of alignment and *k*-mer methods suggest reasonable topological congruence (VanWallendael and Alvarez 2022; Lo *et al.* 2022; Van Etten *et al.* 2023), or no greater incongruence than might be expected from using different alignment-based methods (e.g. Shen *et al.* 2021). This also demonstrates the capacity for *k*-mer -based methods to reconstruct evolutionary history and, when they incorporate models of evolution, be deemed “phylogenetic”.

There are some limitations to alignment-free phylogenetics methods. Unlike conventional alignment-based phylogenetic trees, alignment-free trees do not produce branch lengths with a scale corresponding to geological time, and so one cannot extrapolate the date of divergences. Alignment-free methods also struggle with the reconstruction of deep nodes (Fan *et al.* 2015), which is evident from the *k*-mer trees we present here, although that issue is inherent to all phylogenetics methods (Lanier and Knowles 2015). This may limit the ability of these methods to address questions about inter-relatedness of larger CME clades but should still allow for assessment of more recent divergences.

### Both cargo genes and noncoding cargo content contribute to *k*-mer -based phylogenetic relationships between *Gaeumannomyces Starships*

To explore the extent to which cargo gene content corresponded with the *k*-mer -based phylogenetic relationships, we used twenty *Starships* previously identified from 7 genomes across 3 separate lineages within the genus *Gaeumannomyces,* an understudied member of the *Magnaporthaceae* (Hill *et al.* 2025). These genomes were sequenced from 5 strains of the wheat root pathogen species *G. tritici* (*Gt*) and 2 of the oat root pathogen *G. avenae* (*Ga*). Within the *Gt* strains there is further subdivision of 2 strains belonging to “type A” and 3 to “type B,” 2 distinct genetic lineages present in the species (Palma-Guerrero *et al.* 2021). This division is meaningful, as differences between the 2 types in terms of both virulence and genomic signatures may indicate that these 2 types actually represent cryptic species (Hill *et al.* 2025). As well as being a consistently amassed set of *Starships* for controlled comparison, these *Gaeumannomyces* elements also provided major variability, ranging from ∼32–688 Kbp in total length and containing between 1 and 156 genes (Fig. 2a). It should be noted that 6 of the elements, including both from the *Gt*A strains, were excluded from the first phylogenetic analysis (Fig. 1a) as these elements did not have predicted flanking DRs and so there is some uncertainty as to their exact boundaries. However, we retained them here so as not to exclude potentially biologically meaningful results.

**Fig. 3.**
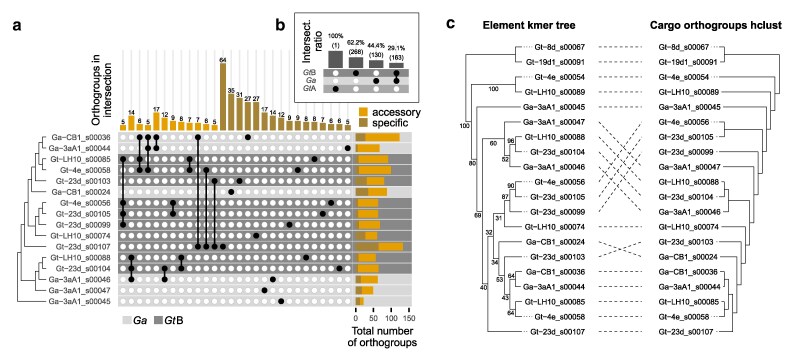
Comparison of cargo gene content similarity across *Gaeumannomyces Starships*. a) An upset plot indicating groups of elements which share at least 5 orthologous genes (accessory), and elements with at least 5 unique cargo genes (specific). Elements are ordered by phylogenetic relationships taken from Supplementary Fig. 2. Total number of cargo orthogroups is shown in the right-hand bar plot with the proportion of accessory and specific cargo genes colored per element. Element rows are colored by host lineage. A representation of all shared accessory orthologous genes is given in Supplementary Fig. 7. b) An upset plot indicating the ratio of orthologous genes shared across lineage/species boundaries. c) A tanglegram comparing the topology of *Gaeumannomyces* elements taken from Supplementary Fig. 2 and a hierarchical clustering of cargo orthologous gene presence-absence.

We found that *Starships* with greater numbers of shared orthologous genes were frequently sister elements or closely related in the *k*-mer tree, for instance, Gt-LH10_s00088, Gt-23d_s00104 and Ga-3aA1_s00046 (Fig. 3a). Most cases of more distantly related elements with high cargo gene sharedness involved the largest and most gene-rich element, Gt-23d_s00107, which incidentally also had one of the highest proportions (48%) of element-specific genes. Hierarchical clustering of cargo orthologous gene content supported these results, with reasonable concordance between the hierarchical clustering and *k*-mer element tree (RF distance 0.47 = 47% differing bipartitions; Fig. 3c) and the most notable deviation between the 2 trees was the divergence of element Gt-23d_s00107. Pairs of closely related elements with evident regions of syntenic cargo genes (Fig. 2a) were often located on different chromosomes, suggesting previous mobilization (e.g. Gt-23d_s00104 and Ga-3aA1_s00046; Ga-CB1_s00036 and Ga-3aA1_s00044; Gt-4e_s00056 and Gt-23d_s00105; Fig. 2b). In contrast, there were also apparently static elements, being closely related and in the same orientation and position within different genomes (e.g. Gt-LH10_s00088 and Ga-3aA1_s00046; Gt-4e_s00058 and Gt-23d_s00103). The question of how similar elements must be to be considered “the same” is also pertinent, as there was one case of closely related elements at different locations within the same host genome, although 1 lacking predicted flanking repeats (Gt-23d_s00105 and Gt-23d_s00099). Elements becoming multi-copy in the genome may arise from mobilization of an ancestral element followed by sexual recombination between 2 hosts with the element in the original and more recent genomic location, respectively (Urquhart *et al.* 2023).

While cargo gene content was evidently a contributing factor to the patterns of *Gaeumannomyces* element relatedness recovered from the *k*-mer -based phylogenies, the nature of a *k*-mer -based approach means that intergenic content within *Starships* must also be implicated. Indeed, repetitive DNA, introns, and presumably other noncoding regions can provide important phylogenetic signals (Lo *et al.* 2022). Here, the only 2 *Gt*A elements found, 1 in each *Gt*A genome, contained a single cargo gene despite being 61 and 73 kbp long. In the larger *k*-mer tree of starfish-predicted elements the *Gt*B and *Ga* elements were closely related to sordariomycete elements from the pathogenic rice blast fungus *Pyricularia oryzae* (syn. *Magnaporthe oryzae*) and a eurotiomycete clade, while the single-gene *Gt*A elements were in a distinct clade more closely related to an element from the sordariomycete species *Sporothrix brasiliensis*, albeit without significant branch support (Supplementary Fig. 2). *S. brasiliensis* is found in soils and vegetation, but is also an opportunistic mammalian pathogen, primarily of humans and cats, due to its temperature-dependent dimorphic lifestyle (Téllez *et al.* 2014). Despite being a similar length (57 kbp) to the *Gt*A elements, the *S. brasiliensis* element contained 19 genes, none of which showed sequence similarity with the single gene found in the *Gt*A elements. This suggests that it was primarily noncoding cargo content that informed *k*-mer -based relationships between the *S. brasiliensis* and *Gt*A elements. The *Gt*A elements were also previously found to have likely undergone repeat-induced point mutation (RIP) (Hill *et al.* 2025). RIP induces transition mutations in repetitive DNA, with a particular bias for C→T mutations targeting CpA dinucleotides, and so RIP-like signatures in genomic sequences manifest as biases in the relative frequencies of dinucleotides (Lewis *et al.* 2009; Hane *et al.* 2015). This raises the question of whether or to what extent signatures of RIP, such as a higher frequency of TpA dinucleotides, influence *k*-mer -based inference of element relationships, especially in cases with extensive intergenic cargo content.

The whole-element *k*-mer trees, captain tree, and the patterns of shared cargo genes indicated that there is no apparent species boundary for *Starship* content between *Gt*B and *Ga.* We found no evidence of similarity with *Gt*A elements, although there was only 1 gene-poor *Gt*A element with which to compare. We see 2 possible scenarios: (1) elements were in the common ancestor of all 3 lineages and lost in *Gt*A or (2) elements are readily exchanged between *Ga* and *Gt*B strains, whether through HGT or interspecific hybridization. Either way, together with the fact that, unlike the other *Gaeumannomyces* elements, the *Gt*A elements were previously found to be subject to element-wide RIP (Hill *et al.* 2025), *Starship* prevalence and divergence may be another symptom of cryptic speciation between *Gt* types. Although *Gt*B and *Ga* elements appear to be closely related, there was an imbalance in how cargo genes were shared, as a higher proportion of *Ga* cargo genes had an orthologue in *Gt*B elements (56%) than *Gt*B cargo genes had in *Ga* elements (38%; Fig. 3b). Additionally, there were differences in how cargo genes were distributed in the genome, with more cargo gene orthogroups only found inside *Ga* elements that had copies integrated into the wider genome in *Gt* strains than the reverse (Supplementary Fig. 8a). In a similar vein, *Ga Starships* broadly had a higher proportion of orthogroups that were only inside the element compared with *Gt*B *Starships* (Supplementary Fig. 8b). Unpicking the differences in relative levels of duplication, sharedness, and location of cargo genes on different *Starships* may be important for determining patterns of inheritance or selection.

### *Gaeumannomyces Starship* cargos harbor a variety of putative plant–fungal interaction genes, but the ToxA gene was notably absent

Most genes previously reported to be common, or notable, in *Starships* (Gluck-Thaler *et al.* 2022) were absent from *Gaeumannomyces Starship* cargos, namely DUF3723, FRE, PLP, and spore-killer (Spok1) genes. There was 1 putative NOD-like receptor (NLR) located on element Gt-23d_s00107 (Fig. 2). The NLR contained a central NACHT domain—the most common nucleotide binding and oligomerization (NOD) domain in fungal NLRs (Daskalov *et al.* 2020)—a WD40 repeat domain, and a sesA N-terminal domain of unknown function (PF17107) that is more common in ascomycete NLRs (Daskalov *et al.* 2020). This sesA-NACHT-WD structure is also found in the NWD3 gene of the model experimental fungus *Podospora anserina* (Daskalov *et al.* 2012). While the function of sesA is not established, other members of the *P. anserina* NWD gene family are involved in heterokaryon/vegetative incompatibility or self/nonself-recognition, which has also been hypothesized to contribute to an innate fungal immune system (Paoletti and Saupe 2009; Uehling *et al.* 2017).

Of particular note was the absence of the necrosis-inducing ToxA effector in the *Gaeumannomyces* cargos, which is located in *Starships* in 3 other wheat pathogens—*Pyrenophora tritici-repentis, Parastagonospora nodorum*, and *Bipolaris sorokiniana* (McDonald *et al.* 2019; Bucknell *et al.* 2024). *Py. tritici-repentis* and *Pa. nodorum* are known to frequently co-infect wheat (Abdullah *et al.* 2020), and *Py. tritici-repentis* and *B. sorokiniana* together form a leaf blight disease complex (Kumar *et al.* 2002). While we could not find information on the potential co-occurrence of *Gaeumannomyces* spp. and other wheat pathogens in the literature, based on their global distributions and the global distribution of the wheat crop, it is highly likely that *Gaeumannomyces* spp. also co-occur with 1 or more of these wheat pathogens (Větrovský *et al.* 2020), which would have provided the opportunity to exchange *Starships.* However, all 3 species containing ToxA reside in a different class, *Dothideomycetes*, in the order *Pleosporales*. At the present time, the lack of ToxA in the *Gaeumanomyces Starships* is consistent with our *k*-mer tree results indicating a host relatedness boundary to *Starship* exchange.

Regarding whether the *Gaeumannomyces Starship* cargos exhibited a core functional role, GO term enrichment analysis of cargo genes reflected high variability as there was no significant enrichment in most elements, although ubiquinone biosynthesis and regulation of translational fidelity were significantly enriched in Ga-3aA1_s00044 and Ga-CB1_s00036, respectively. There were no cargo orthogroups that were core to all elements, but 5 orthogroups were present in at least 50% of the elements (Supplementary Fig. 7). One was predicted to be a carbohydrate-active enzyme (CAZyme) belonging to glycosyltransferase family 2 (GT2; Fig. 2). The GT2 family includes enzymes necessary for the synthesis of chitin (Lairson *et al.* 2008), which is required for the structural integrity of the fungal cell wall (Bowman and Free 2006). A GT2 enzyme has been demonstrated to be required for the disease-causing abilities of the wheat pathogens *Zymoseptoria tritici* and *Fusarium graminearum* (King *et al.* 2017). Expansion and contraction of GT2 CAZyme genes have been shown to be strong predictors of phytopathogenicity and saprotrophy, respectively (Dort *et al.* 2023), but GT2 genes are also expanded in mycorrhizal lineages (Rosling *et al.* 2024), suggesting a key role in both pathogenic and mutualistic plant–fungal interactions. In addition to the prevalent GT2 orthogroup, other CAZymes and CAZyme families were found in various elements: sterol 3β-glucosyltransferase (GT1), glycoside hydrolase (GH) family 33, α-galactosidase (CBM35 + GH27), and glucose-methanol-choline oxidoreductase (AA3_2) in elements Ga-3aA1_s00044 and Ga-CB1_s00036; chitinase (GH18) in Gt-LH10_s00085; and another GT2 CAZyme in Gt-23d_s00099.

Multiple *Gaeumannomyces Starship* cargo genes had BLAST hits to genes in the PHI-base database, which compiles and curates experimentally verified genes implicated in pathogen–host interactions (Urban *et al.* 2025). This included 4 genes in the closely related *P. oryzae* which have been associated with virulence in barley and rice, 2 of which are implicated in calcium signaling and 2 transcription factors, and the previously mentioned GT2 CAZyme which has been associated with virulence of *Zymoseptoria tritici* and *Fusarium graminearum* in wheat leaves and floral spikes, respectively (Table 1). Intriguingly, the chitinase CAZyme cargo gene in element Gt-LH10_s00085 matched a chitinase gene in the mycoparasite *Trichoderma virens* which is associated with its virulence toward the basidiomycete plant pathogen *Rhizoctonia solani*. *Trichoderma* species are known for endophytic colonization of plants, particularly roots, and in some cases can reduce disease via both inducing plant resistance and direct antagonism of other fungi (Harman *et al.* 2004). Two further orthogroups had BLAST hits to CAZyme genes in PHI-base (Xyp1 and PELB/CcpelA); however, as these were not previously flagged during CAZyme annotation (Hill *et al.* 2025) there remains some uncertainty as to their function.

**Table 1.** PHI-base genes with BLAST hits in *Gaeumannomyces Starship* cargos.

PHI-base ID	Gene	Function	Species	Mutant phenotype	Plant–host
PHI:7559	FgGT2	glycosyltransferase	*Fusarium graminearum*	Loss of pathogenicity	*Triticum aestivum*
PHI:2057	MoPLC1	modulator of calcium flux	*Pyricularia oryzae^b^*	Loss of pathogenicity	*Oryza sativa*
PHI:3837	Sre1	iron-sensitive transcription factor	*Bipolaris maydis*	Reduced virulence	*Zea mays*
PHI:2476	CcpelA*^a^*	pectate lyase	*Colletotrichum coccodes*	Reduced virulence	*Solanum lycopersicum*
PHI:222	PELB*^a^*	pectate lyase	*Colletotrichum gloeosporioides*	Reduced virulence	*Persea americana*
PHI:9042	nrps5 (FGSG_13878)	non-ribosomal peptide synthetase	*Fusarium graminearum*	Reduced virulence	*Triticum aestivum*
PHI:6262	FUG1	role in pathogenicity and fumonisin biosynthesis	*Fusarium verticillioides*	Reduced virulence	*Zea mays*
PHI:3315	conx1	Zn^2^Cys^6^ transcription factors	*Pyricularia oryzae^b^*	Reduced virulence	*Oryza sativa*
PHI:3308	cnf1	Zn^2^Cys^6^ transcription factors	*Pyricularia oryzae^b^*	Reduced virulence	*Hordeum vulgare*
PHI:2113	Kin4	Ca^2+^/CAM-dependent serine/threonine protein kinases	*Pyricularia oryzae^b^*	Reduced virulence	*Hordeum vulgare*
PHI:144	CHT42	chitinase	*Trichoderma virens*	Reduced virulence	*Rhizoctonia solani*
PHI:3210	FfSge1	morphological switch regulator	*Fusarium fujikuroi*	Unaffected pathogenicity	*Oryza sativa*
PHI:1603	GzOB044	transcription factor	*Fusarium graminearum*	Unaffected pathogenicity	*Triticum*
PHI:1377	GzC2H040	transcription factor	*Fusarium graminearum*	Unaffected pathogenicity	*Triticum*
PHI:6639	Modnm3	dynamin	*Pyricularia oryzae^b^*	Unaffected pathogenicity	*Oryza sativa*
PHI:6613	Mocapn14	calpain	*Pyricularia oryzae^b^*	Unaffected pathogenicity	*Oryza sativa*
PHI:124206	Xyp1 (Uv8b_02447)	cell wall degrading enzymes	*Ustilaginoidea virens*	Increased virulence	*Oryza sativa*

^a^Pectate lyases CcpelA and PELB matched to the same orthogroup.

^b^ *Pyricularia oryzae* = *Magnaporthe oryzae*.

Also of note is that none of the biosynthetic gene clusters (BGCs) previously identified in the *Gaeumannomyces* genomes were present in any *Starships*, but 2 cargo genes had hits to PHI-base genes implicated in secondary metabolite synthesis in *Fusarium* species, namely nrps5 and FUG1. The latter is involved in fumonisin (FUM) synthesis in *Fusarium verticillioides* (Ridenour and Bluhm 2017), but is located on a separate locus to the FUM gene cluster, suggesting that it may play a regulatory role, as biosynthesis transcription factors can frequently be located outside of contiguous BGCs (Kwon *et al.* 2021). FUG1 was also previously found to have orthologs across *Ascomycota*, including in *Gt* (Ridenour and Bluhm 2017). The non-ribosomal peptide synthetase nrps5 gene is located alongside nrps9 in an 8-member BGC cluster in *Fusarium* species, which produces fusaoctaxin A and is essential to virulence of *F. graminearum* in wheat (Jia *et al.* 2019). However, none of the genes surrounding the nrps5-like gene in the *Gaeumannomyces* elements showed similarity to the other nrps5/9 cluster members. We also found an uncharacterized candidate secreted effector protein (CSEP) gene in 1 element (Ga-CB1_s00036). Intriguingly, this CSEP was located within a region that was highly syntenic with another element (Ga-3aA1_s00044) but the CSEP was not present in that second element (Fig. 2), underlining the dynamism of *Starship* cargos.

## Conclusions

Here, we provide evidence of a difference in evolutionary history between *Starship* elements in their entirety vs their captain genes. This raises the question: is it more important to define *Starships* by their mode of mobilization—i.e. the tyrosine recombinase captain gene—or the cargo of genes and noncoding/repetitive content mobilized? The answer to that question will depend on the context in which the question is asked, namely, whether the inquiry at hand is to understand the mechanism of transposition, or to understand how elements and their cargos evolve and impact host fitness. Whole-element relationships are easily assessed using *k*-mer -based phylogenetic methods, which have revealed previously hidden signals corresponding to host taxonomy. These methods also allow us to assess relationships including “degraded” elements where captains and/or DRs/TIRs have been lost. By accounting for the composition of *Starships* without being hampered by alignment issues caused by repeats, indels, duplications, rearrangements and inversions, or lack of available sequences in general, *k*-mer -based phylogenetic methods can help to refine the existing haplotype-based classification of CMEs. Beyond informing classification, this new approach could also provide context and new insights to address fundamental outstanding questions regarding *Starships* and other CMEs, such as the evolutionary origins of elements, the prevalence of HGT, and the role of elements in the host genome.

## Supplementary Material

jkaf082_Supplementary_Data

## Data Availability

All original data sources used in this study are cited in the text. Analysis scripts are available at https://github.com/Rowena-h/StarshipTrees. Supplemental material available at G3 online.
