# Supplementary material for: Starship giant transposable elements cluster by host taxonomy using k-mer-based phylogenetics
Source: G3 (Bethesda). 2025 Apr 11;15(6):jkaf082. doi: 10.1093/g3journal/jkaf082 (PMC12134995; doi:10.1093/g3journal/jkaf082)
Supplement: jkaf082_Supplementary_Data [file jkaf082_supplementary_data.pdf]

Starship giant transposable elements cluster by host taxonomy using kmer-based phylogenetics

Supplementary material

Rowena Hill[1], Daniel Smith[2,†], Gail Canning[3], Michelle Grey[1], Kim Hammond-Kosack[3], and Mark McMullan[1]

[1]*Earlham Institute, Norwich, Norfolk, NR4 7UZ, UK*
[2]*Intelligent Data Ecosystems, Rothamsted Research, Harpenden, Hertfordshire, AL5 2JQ, UK*
[3]*Protecting Crops and the Environment, Rothamsted Research, Harpenden, Hertfordshire, AL5 2JQ, UK*
[†]*Present address: Department of Computational and Systems Biology, John Innes Centre, Norwich, Norfolk, NR4 7UH, UK*

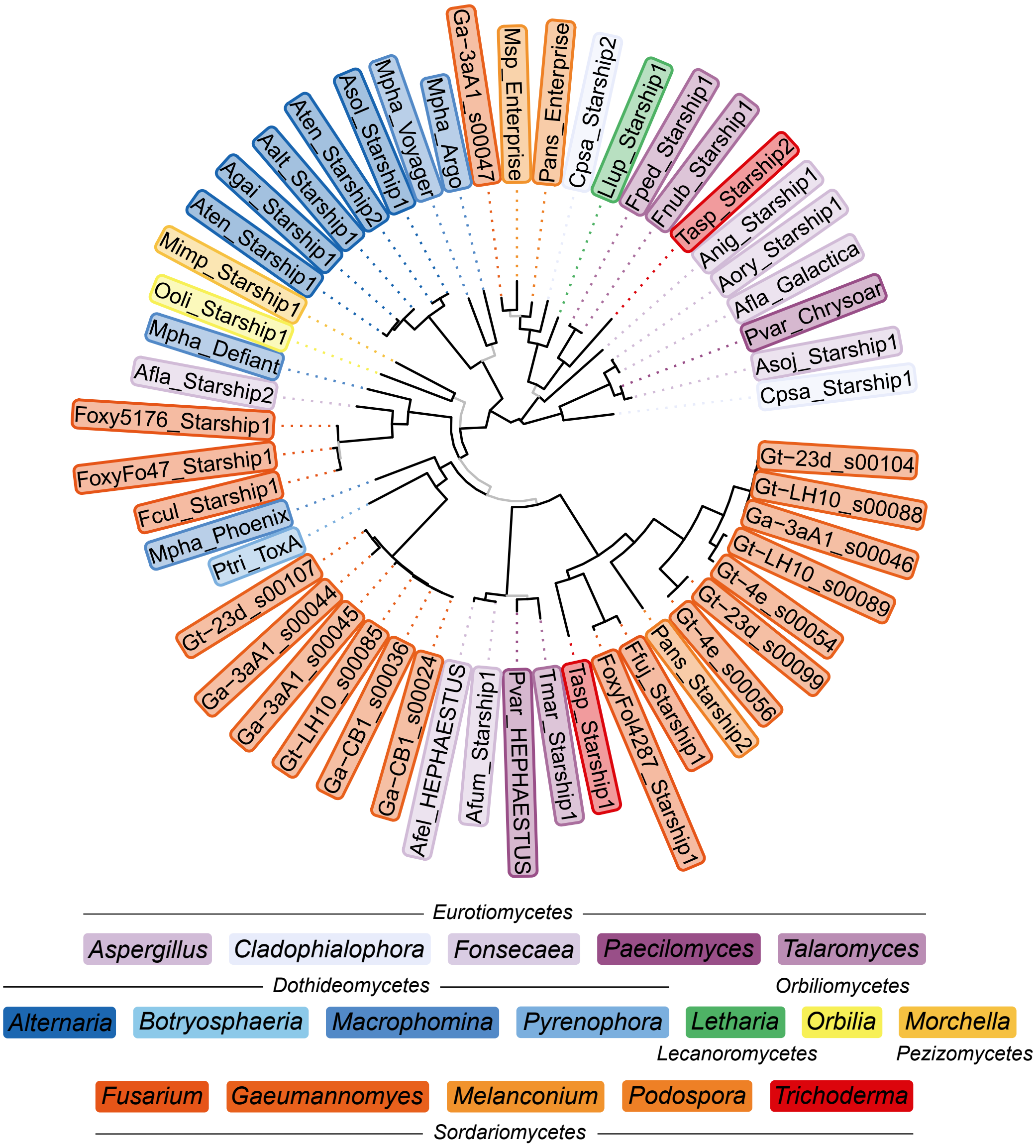

Supplementary Fig. 1 An unrooted maximum likelihood (ML) phylogenetic tree of captain genes from 53 *Starships* – 39 curated elements (Gluck-Thaler, Ralston, *et al.*, 2022; Gluck-Thaler and Vogan, 2024) and 14 predicted by starfish (Hill *et al.*, 2025). Grey branches indicate bootstrap support < 70. Tips are coloured by genus.

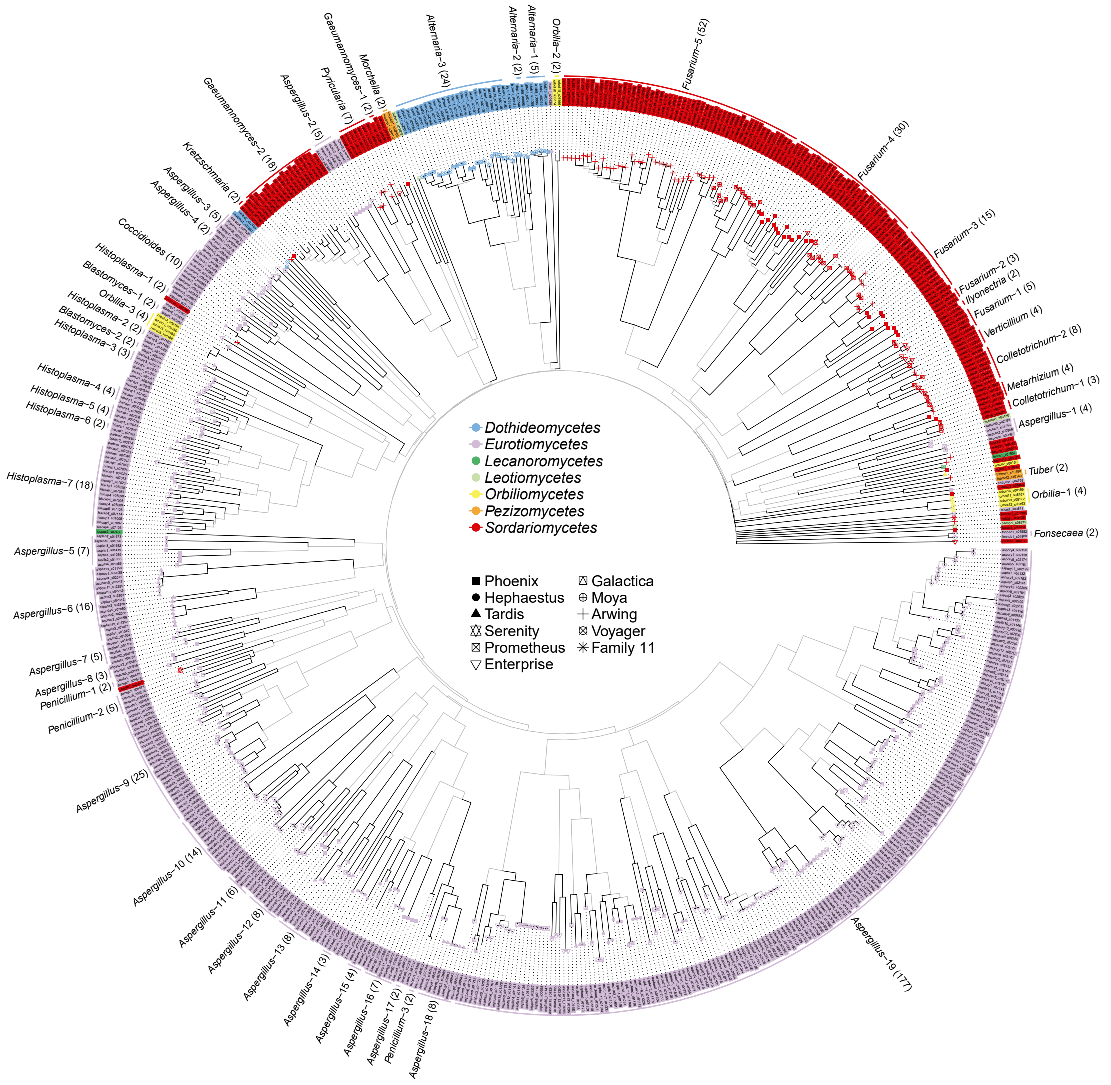

Supplementary Fig. 2 An unrooted kmer-based phylogenetic tree of 617 *Starships* predicted with starfish (Gluck-Thaler and Vogan, 2024), with grey branches indicating bootstrap support < 70. Genus-level monophyletic clades are highlighted and labelled, with the number of elements in each clade shown in brackets. Clades and tips are coloured by host taxonomic class. Previously determined family classification based on phylogenetic relationships of captain genes (Gluck-Thaler and Vogan, 2024) are indicated by shapes on tip points.

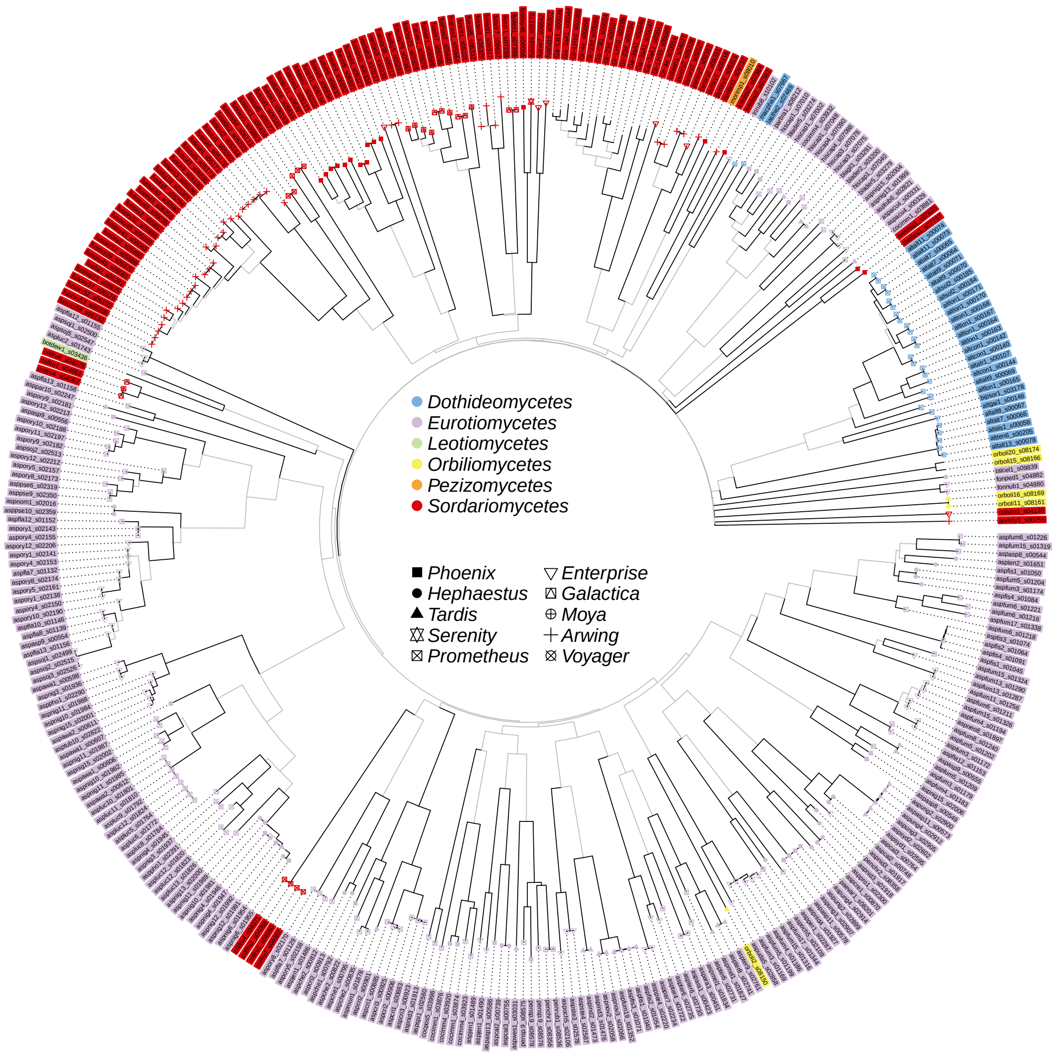

Supplementary Fig. 3 An unrooted kmer-based phylogenetic tree of 357 *Starships* predicted with starfish (Gluck-Thaler and Vogan, 2024), filtered from the dataset used in Supplementary Fig. 2 to only include elements with predicted flanking direct repeats. Grey branches indicate bootstrap support < 70. Clades and tips are coloured by host taxonomic class. Previously determined family classification based on phylogenetic relationships of captain genes (Gluck-Thaler and Vogan, 2024) are indicated by shapes on tip points.

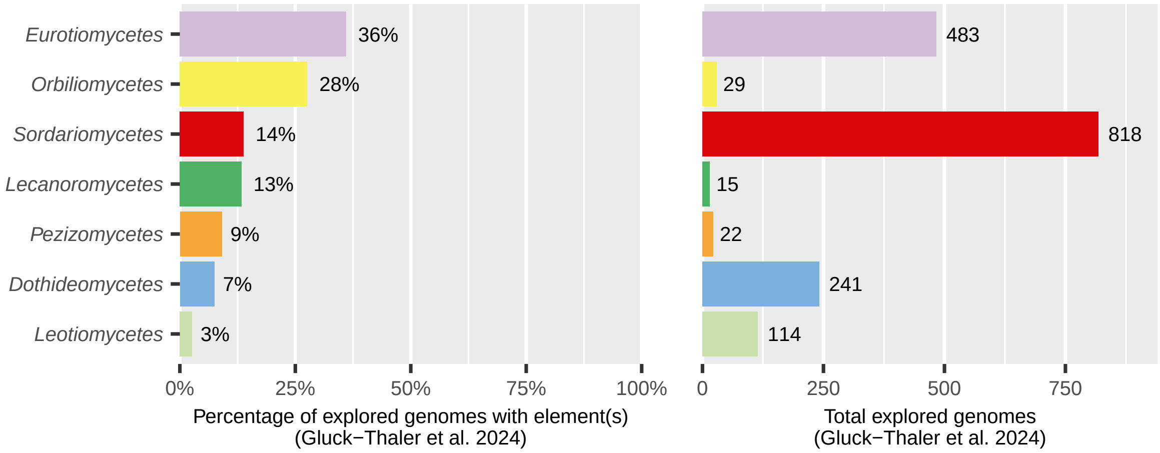

Supplementary Fig. 4 The proportion of genomes in each host taxonomic class containing *Starships* from the Gluck-Thaler and Vogan (2024) dataset.

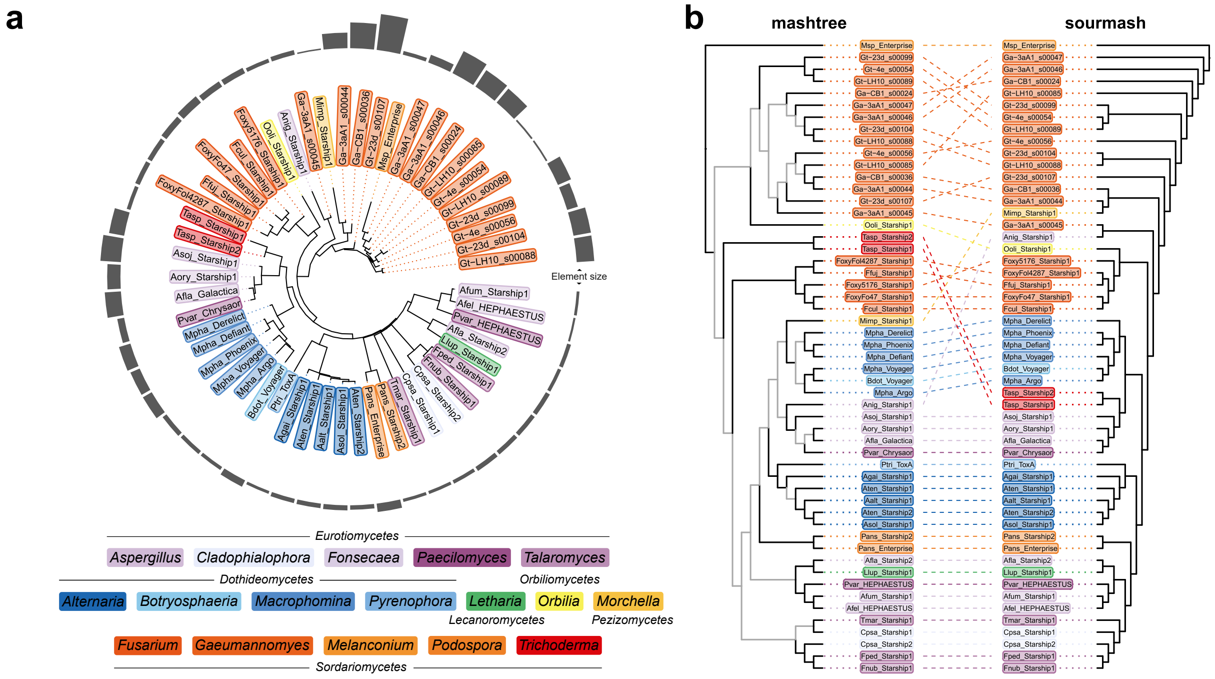

Supplementary Fig. 5 a) An unrooted kmer-based phylogenetic tree of 53 Starships – 39 curated elements from 33 *Pezizomycotina* species (Gluck-Thaler, Ralston, *et al.*, 2022; Gluck-Thaler and Vogan, 2024) and 14 predicted by starfish from *Gaeumannomyces* species (Hill *et al.*, 2025), produced from an average nucleotide identity (ANI) distance matrix generated with sourmash (Irber *et al.*, 2024). Tip points are coloured by genus and the outer ring indicates total element length. b) A tanglegram comparing the topology of the Mashtree tree in Fig. 1a and the sourmash tree. Both trees are arbitrarily rooted with the Msp_Enterprise element. Grey branches for the Mashtree tree indicate bootstrap support < 70.

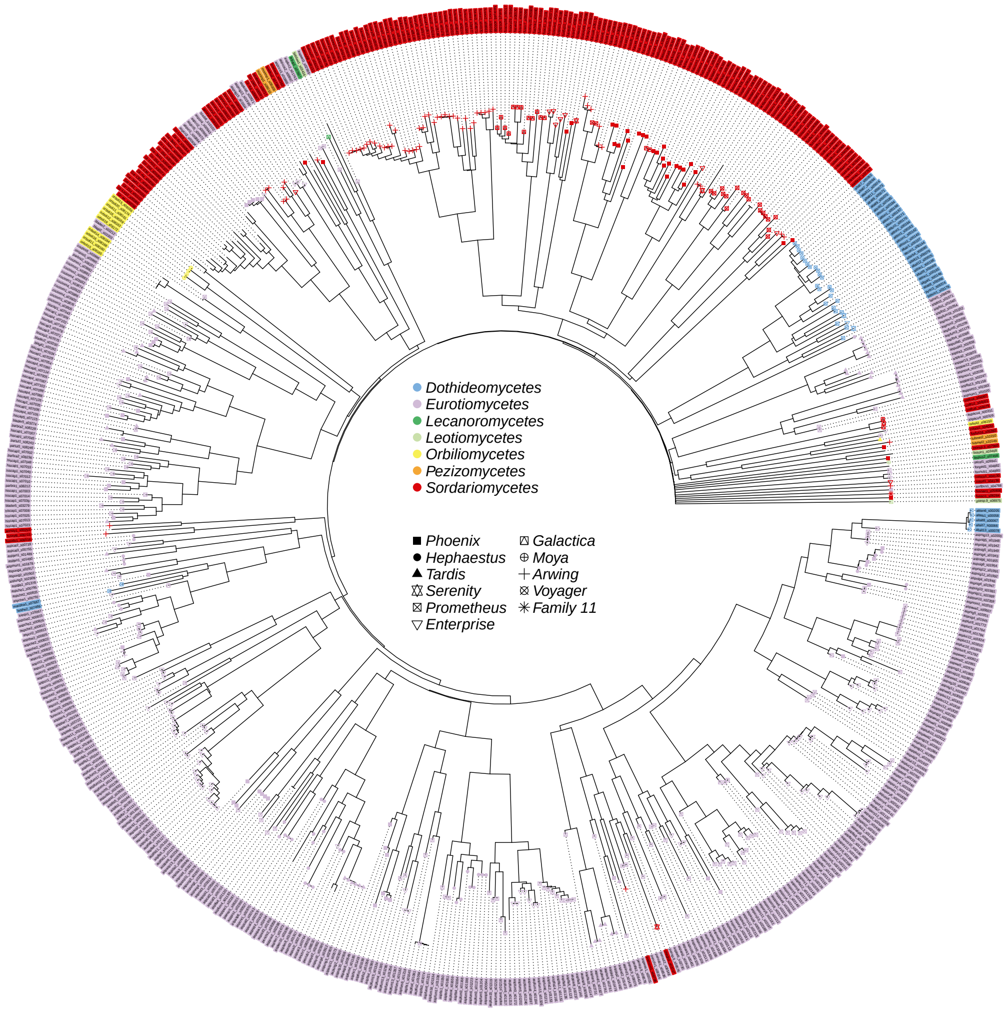

Supplementary Fig. 6 An unrooted kmer-based phylogenetic tree of 617 *Starships* predicted with starfish (Gluck-Thaler and Vogan, 2024), produced from an average nucleotide identity (ANI) distance matrix generated with sourmash (Irber *et al.*, 2024). Tips are coloured by host taxonomic class. Previously determined family classification based on phylogenetic relationships of captain genes (Gluck-Thaler and Vogan, 2024) are indicated by shapes on tip points.

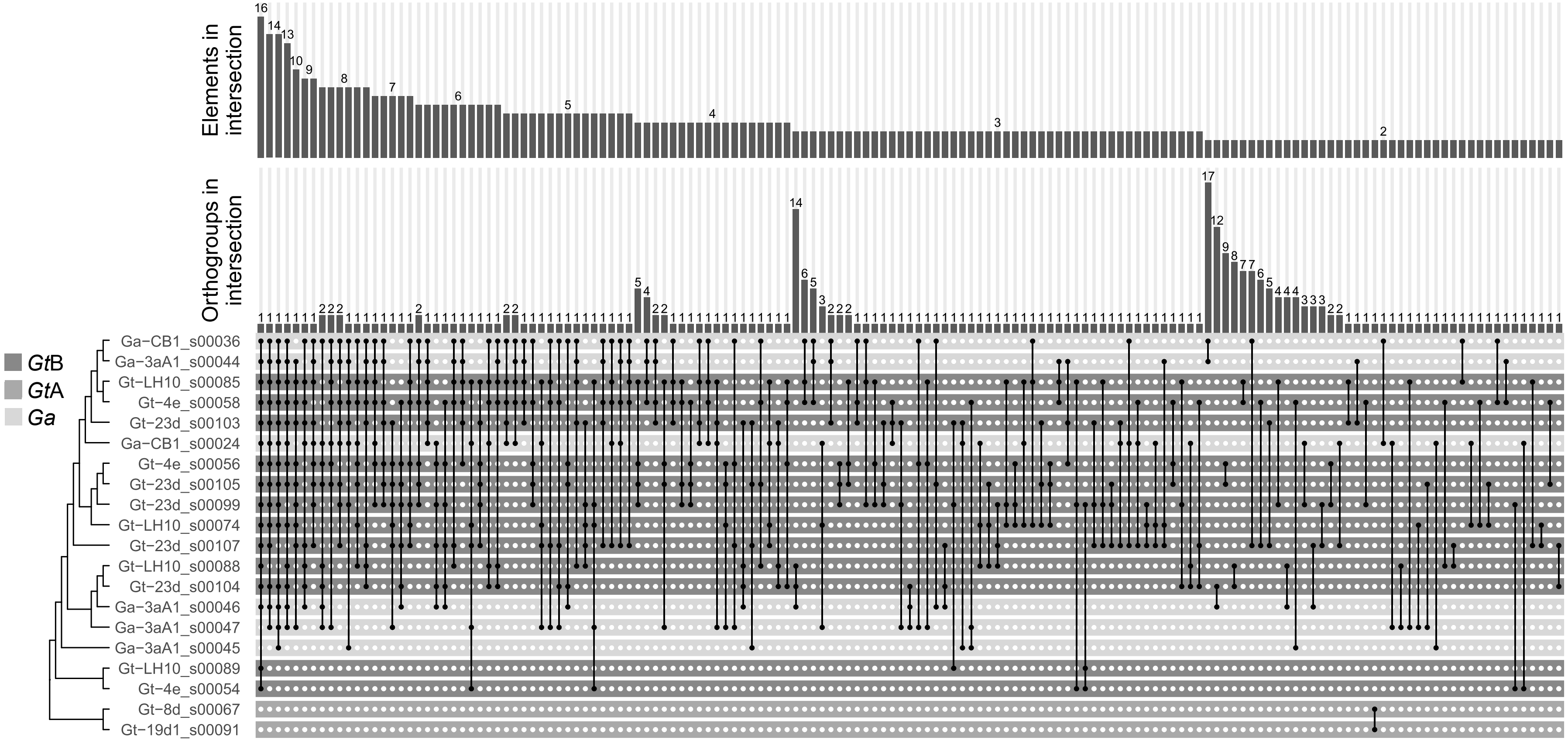

Supplementary Fig. 7 An upset plot indicating how accessory cargo orthogroups are shared across elements. Elements are ordered by phylogenetic relationships taken from Supplementary Fig. 2, with rows coloured by host lineage. Intersections are ordered from most shared to least shared (see upper bar plot).

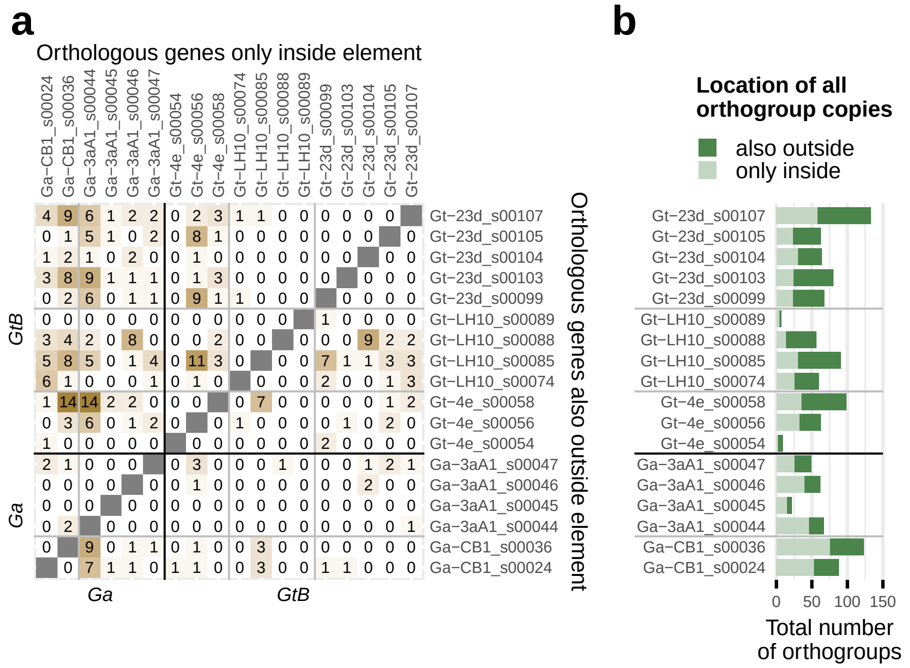

Supplementary Fig. 8 a) A grid showing the number of cargo orthologous genes found only inside versus also outside two given elements. Elements are grouped by host lineage. b) The proportion of total cargo orthogroups that are found only inside or also outside each element.

Gluck-Thaler E, Ralston T, Konkel Z, Ocampos C G, Ganeshan V D, Dorrance A E, Niblack T L, Wood C W, Slot J C, Lopez-Nicora H D, and Vogan A A. 2022. Giant *Starship* Elements Mobilize Accessory Genes in Fungal Genomes. Mol Biol Evol. 39(5):msac109. doi: 10.1093/molbev/msac109.

Gluck-Thaler E and Vogan A A. 2024. Systematic identification of cargo-mobilizing genetic elements reveals new dimensions of eukaryotic diversity. Nucleic Acids Res.1–18. doi: 10.1093/nar/gkae327.

Hill R, Grey M, Olivera Fedi M, Smith D P, Canning G, Ward S J, Irish N, Smith J, McMillan V, Hammond J, Osborne S.-J, Reynolds G, Smith E, Chancellor T, Swarbreck D, Hall N, Palma-Guerrero J, Hammond-Kosack K E, and McMullan M. 2025. Evolutionary genomics reveals variation in structure and genetic content implicated in virulence and lifestyle in the genus *Gaeumannomyces*. BMC Genomics 26:239. doi: 10.1186/s12864-025-11432-0.

Irber L, Pierce-Ward N T, Abuelanin M, Alexander H, Anant A, Barve K, Baumler C, Botvinnik O, Brooks P, Dsouza D, Gautier L, Hera M R, Houts H E, Johnson L K, Klötzl F, Koslicki D, Lim M, Lim R, Nelson B, Ogasawara I, Reiter T, Scott C, Sjödin A, Standage D, Swamidass S J, Tiffany C, Vemuri P, Young E, and Brown C T. 2024. sourmash v4: A multitool to quickly search, compare,and analyze genomic and metagenomic data sets. Journal of Open Source Software 9(98):6830. doi: 10.21105/joss.06830.
